# Cross‐Modal Transfer Effects of the Go/No‐Go Training With Visual Stimuli

**DOI:** 10.1002/brb3.70309

**Published:** 2025-02-09

**Authors:** Maiko Hori, Sho Kojima, Hideaki Onishi

**Affiliations:** ^1^ Department of Physical Therapy Niigata University of Health and Welfare Niigata Japan; ^2^ Institute for Human Movement and Medical Sciences Niigata University of Health and Welfare Niigata Japan

**Keywords:** cross‐modal transfer, Go/No‐go task, tactile modality, visual modality

## Abstract

**Introduction:**

Motor task performance guided by one sensory modality (e.g., visual stimuli) can be improved by training; however, whether this training can also improve performance on the same task guided by another sensory modality remains uncertain (e.g., tactile stimuli). This study examined the effects of Go/No‐go task training using visual stimulus cues on Go/No‐go task performance using tactile cues and whether training also influences the dominance of the sensory modality.

**Methods:**

Go/No‐go tasks and a temporal order judgment (TOJ) task were performed by 24 right‐handed, healthy adults on days 1 and 5 of the 5‐day experiment. Furthermore, a subpopulation (the training group) practiced the Go/No‐go task with visual stimulus cues on days 2–4, whereas the remaining control group did not practice the task.

**Results:**

The training group demonstrated significantly reduced reaction times (RTs) on both the visual and tactile Go/No‐go tasks by day 5, whereas the control group demonstrated significantly reduced RTs only on the tactile Go/No‐go task. The RT change was also significantly greater in the training group than in the control group for both modalities. Conversely, no significant change in the TOJ for visual and tactile stimuli was observed between the groups.

**Conclusion:**

These results indicate that motor training with visual guidance can improve performance on the same task guided by tactile stimuli, possibly due to neuroplastic changes in the multimodal association cortices.

## Graphical abstract

Visual modality training reduced reaction time. The rate of change was greater in the visual modality training group.

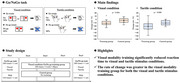

## 1 Introduction

Humans construct a coherent representation of the external environment by integrating sensory information from the visual, auditory, tactile, gustatory, olfactory, and other sensory pathways (Speed and Brybaert 2022). The discrimination capacity and processing speed of a specific modality can be improved by training (sensory learning). Several previous studies have demonstrated such improvements using the Go/No‐go task in which participants respond to one specific stimulus (Go stimulus) but not another (No‐go stimulus) (Benikos, Johnstone, and Roodenrys 2013; Bodmer and Beste 2017; Sugawara et al. 2013; Yamashiro et al. 2021). Benikos, Johnstone, and Roodenrys (2013) measured reaction times (RTs) in a Go/No‐go task with visual Go and No‐go stimuli and reported that RTs to Go stimuli were progressively reduced with an increasing number of trials. Furthermore, the duration from stimulus presentation to the onset of the electromyogram (EMG) response (motor response) was significantly reduced 3 days post‐training (Sugawara et al. 2013). Furthermore, RT was significantly shortened 6 days post‐training in a reaction task using tactile stimuli, and the training effect was sustained up to 6 months thereafter (Cohen, Aisenberg, and Henik 2018). Therefore, reaction task training using one specific modality can significantly improve the task performance (Benikos, Johnstone, and Roodenrys 2013; Cohen, Aisenberg, and Henik 2018; Sugawara et al. 2013).

Each sensory modality induces neural activity in specific brain regions (primary sensory cortices) and multimodal regions responsible for sensory integration (Downar et al. 2000; Man et al. 2015; So, Kim, and Kim 2020). For instance, Downar et al. (2000) revealed that the spindle gyrus (visual cortex), the secondary somatosensory cortex (tactile cortex), and the superior temporal gyrus (auditory cortex) are activated by inputs of the corresponding sensory modality, as detected using functional magnetic resonance imaging (fMRI). Furthermore, activity levels were elevated in the bilateral temporoparietal junction and right anterior insular cortex in response to all three modalities, indicating that these areas are multimodal (Downar et al. 2000). These multimodal regions allow for the transfer of modality‐specific learning. Furthermore, other multimodal regions may integrate sensory information with motor plans or proprioceptive feedback for learning motor tasks guided by sensory stimuli. For instance, the typical Go/No‐go task requires motor control functions to execute and inhibit movements in response to Go and No‐go stimuli, respectively, and several regions required for such sensory‐guided motor task performance have been identified (Aron 2007; Chambers, Garavan, and Bellgrove 2009; Jahfari et al. 2011; Osada et al. 2019), including the inferior frontal gyrus (IFG) and the presupplementary motor area (preSMA) (Osada et al. 2019). Activity and neuroplastic changes in these multimodal regions may allow for training effects that cross‐sensory modalities. In other words, improvements in motor task performance guided by one sensory modality may be observed in the same task guided by another modality due to the activity in multimodal regions. Therefore, this hypothesis was tested at the functional level.

Temporal order judgment (TOJ) is commonly used to evaluate the capacity for the detection of temporal asynchrony between different sensory modalities (Harrar and Harris 2008; Spence et al. 2003). A TOJ task presents two types of sensory stimuli separated by various time intervals and requires the participant to determine which was presented first. Judgment accuracy allows the assessment of superiority (dominance) between the two modalities by calculating the point of subjective simultaneity (PSS) (Vroomen et al. 2004). Previous studies using TOJ have reported that sensory training changes PSS (Harrar and Harris 2008; Vroomen et al. 2004). For instance, Vroomen et al. (2004) reported that PSS changes after several minutes of exposure to temporally asynchronous auditory and visual stimuli (Vroomen et al. 2004).

Based on a previous study on brain function, this present study focused on the behavioral level of the cross‐modal transfer effects. If cross‐modal transfer effects could be improved after one‐modality training, the range of training methods would be expanded, thus providing very useful information. Therefore, this study aimed to determine the effects of Go/No‐go task training with a visual modality on task performance and modality dominance with a tactile modality.

## 2 Materials and Methods

### 2.1 Participants

Twenty‐four healthy right‐handed adults (age: 21.1 ± 0.8 years; 8 males and 16 females) without a history of neurological or psychiatric disorders were recruited as study participants. All participants provided informed consent after a full explanation of the study methods and goals. This study was conducted in compliance with the Declaration of Helsinki principles, and the study protocol was approved by the Ethics Committee of Niigata University of Health and Welfare (18235).

### 2.2 Go/No‐Go Task

**FIGURE 1:**
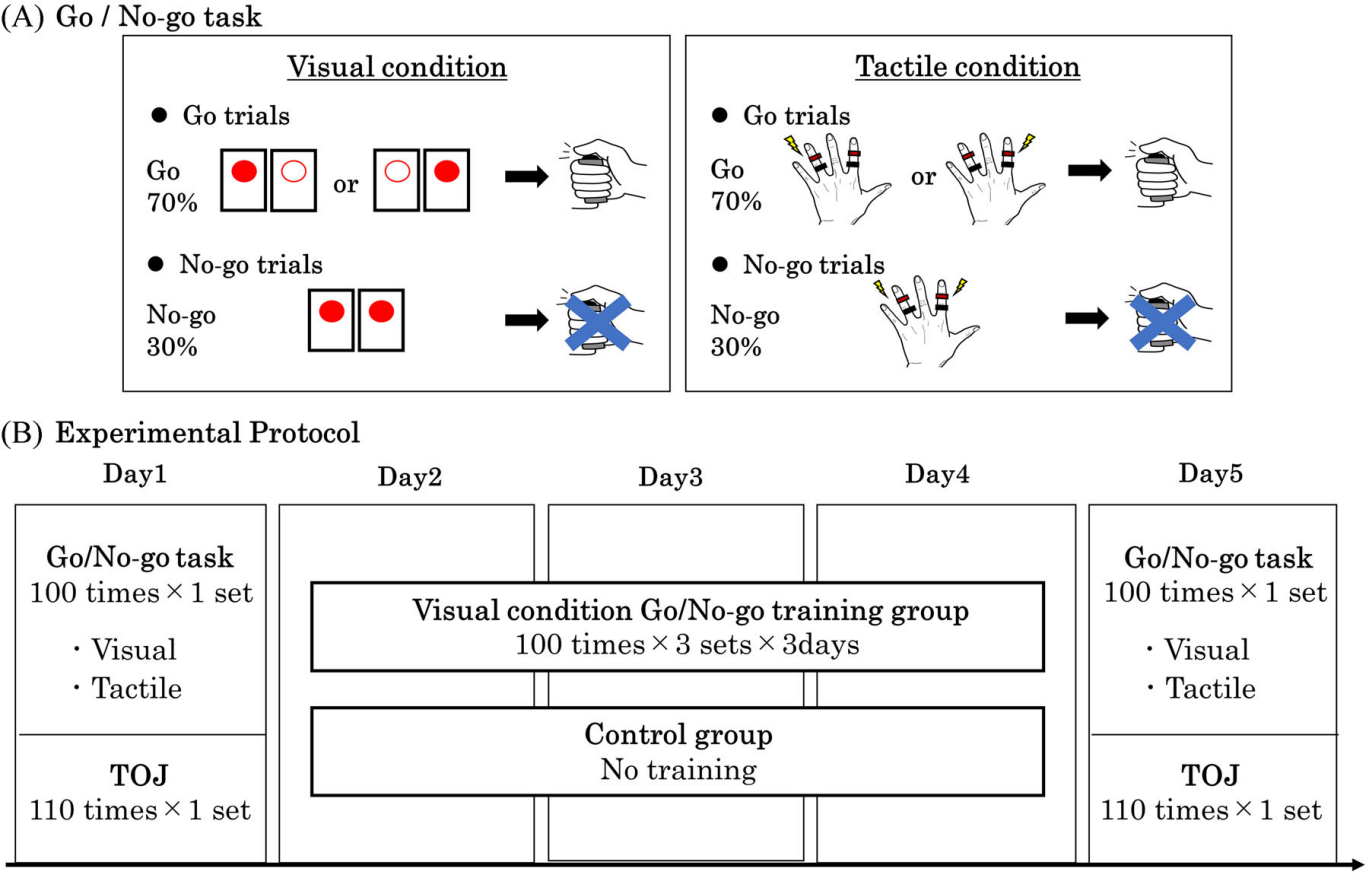
(A) Illustration of the Go/No‐go task. The subjects pressed a button as quickly as possible when the right or left stimulus was presented and did not press the button when both stimuli were presented simultaneously. Red lights were presented in the visual condition, and electrical stimuli were applied to the left index and ring fingers in the tactile condition. For each condition, 70% of the trials presented Go stimuli and 30% presented No‐go stimuli. (B) Schematic illustration of the experimental procedure. The experiment was conducted over 5 days. On day 1, one session of the visual Go/No‐go task and one session of the tactile Go/No‐go task (100 trials per session) as well as one TOJ task session were performed. On days 2–4, the training group practiced three sets of the visual Go/No‐go task, while the control group did not. On day 5, both groups performed one visual and one tactile Go/No‐go task session and the TOJ task as on day 1.

A Go/No‐go task was designed to assess the effects of training using one sensory modality on performance using the same or a different sensory modality (Figure 1A). Two stimulus conditions were used: visual and tactile. The red light output from two visual stimulators was used to guide the Go or No‐go decisions (Takei Scientific Instruments Co., Ltd., Niigata, Japan). In brief, participants were instructed to press a button as quickly as possible when the right or left visual stimulus (Go stimulus) was presented for 50 ms but to remain at rest without movement when both visual stimuli (No‐go stimulus) were presented at the same duration (Figure 1A). In the tactile version of the Go/No‐go task, an electrical stimulator (Nihon Kohden Co., Ltd., Tokyo, Japan) connected to ring electrodes on the fingers was used to cue the Go and No‐go responses. Briefly, one electrode pair was placed on the proximal aspect of the proximal interphalangeal joint and the proximal aspect of the distal interphalangeal joint of the left index finger, whereas the other pair was positioned at the same sites on the left ring finger. The stimulus intensity was set to approximately twice the sensory threshold, with the stimulus presentation time set to 1 ms. Participants were instructed to press the button as quickly as possible when presented with tactile stimulation of the index or ring finger (Go stimulus) and to remain at rest without movement when presented with stimulation to both fingers (No‐go stimulus) (Figure 1A). For both visual and tactile Go/No‐go tasks, trials were presented every 2000–3000 ms, and each session included 70 Go and 30 No‐go trials presented randomly.

### 2.3 TOJ Task

This study used a TOJ task to assess the superiority (dominance) between sensory modalities. The stimulus conditions were the same as in the Go/No‐go task; however, the visual stimulus in the TOJ was presented on the right side only, whereas the tactile stimulus was presented only on the left index finger. Participants were instructed to press a button as quickly as possible when they perceived that the tactile stimulus preceded the visual stimulus. This study used 11 interstimulus intervals (ISIs): −200, −150, −100, −50, −25, 0, 25, 50, 100, 150, and 200 ms. For example, when ISI = 200 ms, the tactile stimulus was presented 200 ms earlier, and when ISI = −200 ms, the visual stimulus was presented 200 ms earlier. Ten trials were performed at each ISI, for a total of 110 trials. The trial interval was 2000 ms.

### 2.4 Experimental Procedure

**FIGURE 2:**
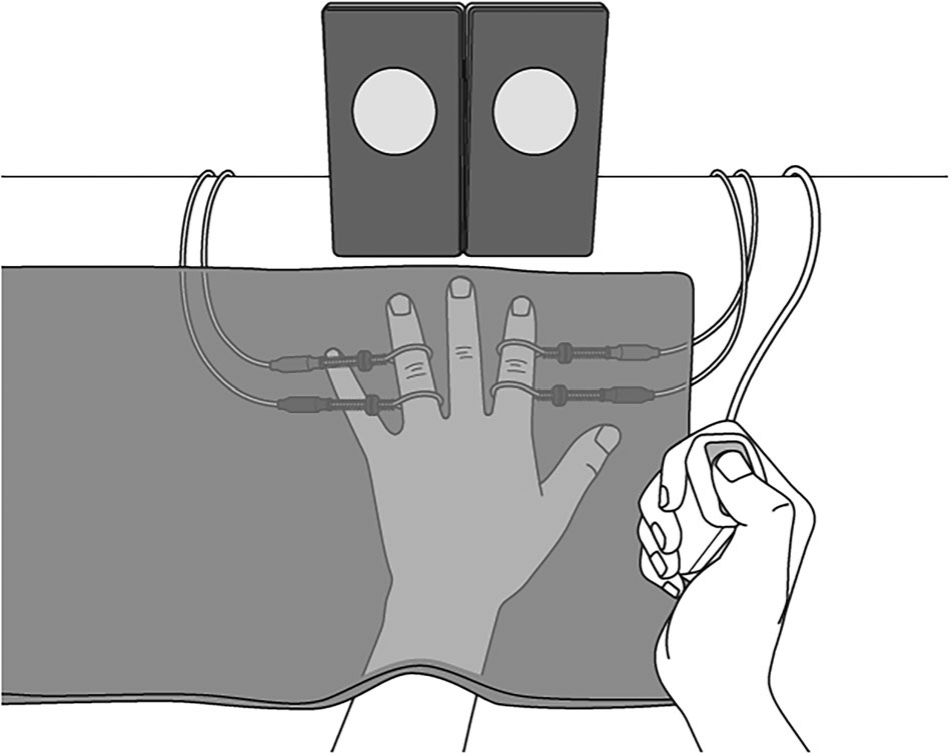
Illustration of the measurement environment. Participants sat in a chair with the visual stimulator in the center. The left hand was placed on a desk, with the index finger on the right visual stimulator and the ring finger on the left visual stimulator, as close as possible to their extensions. In the tactile Go/No‐go trials, the left hand was covered with a cloth so that the stimulation sites could not be seen. The right hand was used to hold the button under both conditions.

The experimental procedure is shown in Figure 1B. The experiment was conducted for 5 days. Participants sat comfortably in a chair during all experiments. The left hand was placed on a desk in front of the participant with the index finger on the right visual stimulator and the ring finger on the left visual stimulator as close as possible to each extension. In the tactile Go/No‐go trials, a cloth was placed over the hand so that the stimulation site could not be observed (Figure 2). First, the stimulus intensity was set for each participant, and then the task was performed. Participants performed one set each of the visual Go/No‐go task and tactile Go/No‐go task, and then the TOJ on day 1 for preintervention (baseline) assessment. Participants were then divided into two groups on days 2–4. The training group (12 participants, age: 21.3 ± 0.7 years) practiced three sets of the Go/No‐go task with visual stimulation in the same measurement environment on day 1, whereas the control group (12 participants, age: 20.9 ± 0.3 years) did not practice the task during these 3 days. All participants performed one set each of the Go/No‐go tasks with visual and tactile stimuli and the TOJ task on day 5 as a postintervention assessment using the same procedures as on day 1.

### 2.5 Data Analysis

The median RT for 70 Go trials pre‐ and postintervention (training) and the number of errors (Go on No‐go trials and No‐go on Go trials, respectively) were calculated and compared between the control and training groups. RTs of ≤150 ms were excluded as outliers based on previous studies (Pierrieau et al. 2021). Trial rejections in this study were 0.78% (all trial average).

The probability of perceiving tactile stimuli first during the TOJ task was plotted on the vertical axis against ISI on the horizontal axis, and the relationship was examined by generalized linear regression using MATLAB 2021 (MathWorks, USA). The PSS, defined as the ISI at which stimuli were judged as simultaneous (in ms), was obtained from the regression line.

### 2.6 Statistical Analysis

All statistical analyses were conducted using SPSS version 24 (IBM, USA). The median values were calculated and compared between each pre‐ and post‐training using the nonparametric Wilcoxon signed‐rank test. The number of errors for one participant was excluded from the analysis as an outlier. The change in RT between day 1 and day 5 {(RT on day 5 − RT on day 1) / (RT on day 1) × 100} was calculated for each condition and compared between the training and control groups using the Wilcoxon rank‐sum test. The probability of PSS first was compared between each pre‐ and post‐training using the Wilcoxon signed‐rank test. The significance level was set at *p* < 0.05 for all tests.

## 3 Results

### 3.1 Go/No‐Go Task

**FIGURE 3:**
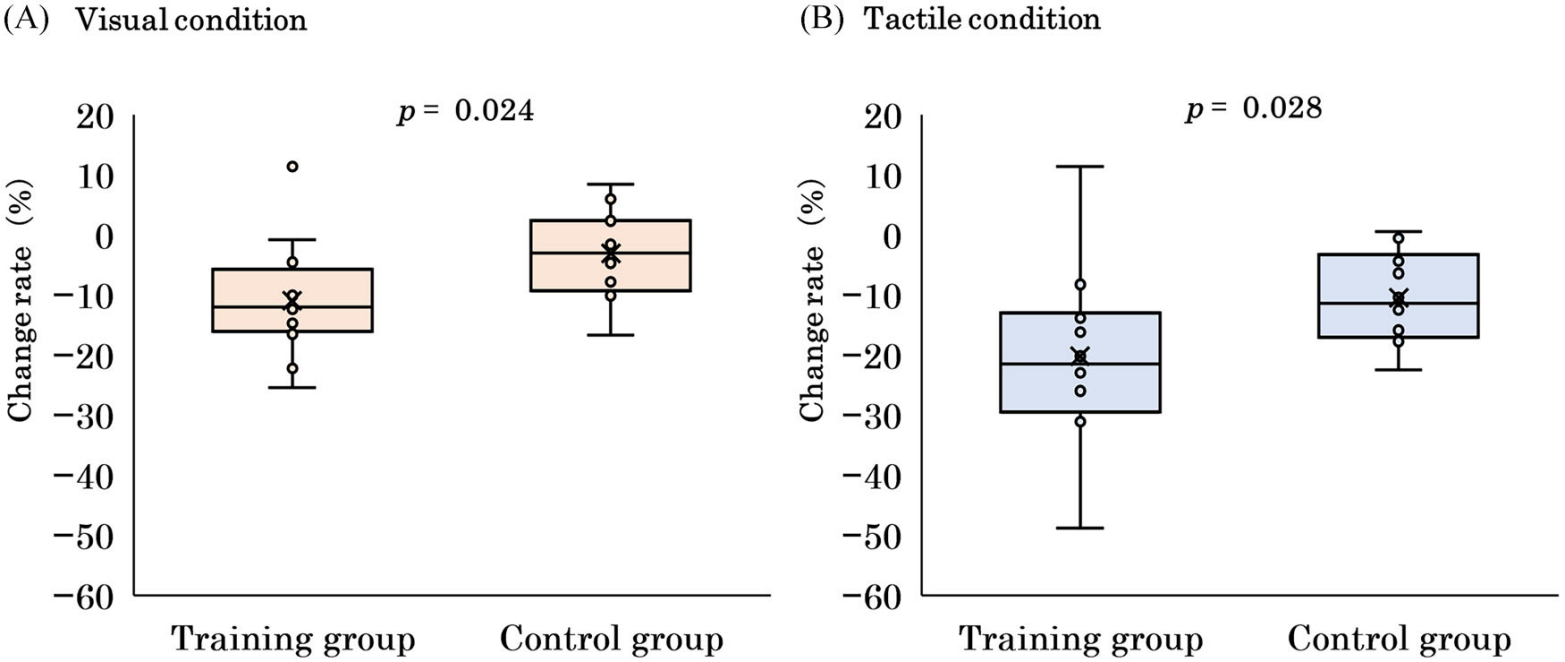
Comparison of the RT change (%) on day 5 versus day 1 between the groups. The change in RT was greater in the training group than in the control group for both the visual (A) and tactile conditions (B). Error bars represent the quartile range. The symbols (open circles) are individual data points.

The median RTs and the number of errors obtained before and after the intervention are shown in Table 1. RTs for the visual Go/No‐go task were significantly shorter on day 5 than on day 1 in the training group (*p* = 0.007, *r* = 0.450) but not in the control group (*p* = 0.182, *r* = 0.240). Conversely, RTs for the tactile Go/No‐go task were significantly shorter in both the training (*p* = 0.004, *r* = 0.470) and control groups (*p* = 0.003, *r* = 0.480). Furthermore, the change in RT was greater in the training group than in the control group for both the visual (*p* = 0.024, *r* = 0.420) and tactile conditions (*p* = 0.028, *r* = 0.410) (Figure 3). The number of No‐go errors on the visual Go/No‐go task was also significantly lower by day 5 in both the training (*p* = 0.028, *r* = 0.410) and control groups (*p* = 0.032, *r* = 0.400). Conversely, the number of Go and No‐go errors on the tactile Go/No‐go task did not differ in either the training or control groups.

**TABLE 1:** Reaction times (RTs) and number of errors on the visual and tactile Go/No‐go tasks.

			Day 1	Day 5	*p*	*r*
Visual	RT	Training group (*n* = 12)	272.5 ± 27.4	242.4 ± 33.5	0.007**	0.450
		Control group (*n* = 12)	273.6 ± 29.0	264.3 ± 26.5	0.182	0.240
	Go Errors	Training group (*n* = 12)	0.0 ± 0.0	0.0 ± 0.0	1.000	—
		Control group (*n* = 12)	0.2 ± 0.4	0.0 ± 0.0	0.157	0.280
	No‐Go Errors	Training group (*n* = 12)	4.8 ± 3.2	3.3 ± 3.1	0.028*	0.410
		Control group (*n* = 12)	3.8 ± 2.2	2.2 ± 1.8	0.032*	0.400
Tactile	RT	Training group (*n* = 12)	403.3 ± 85.8	317.5 ± 67.4	0.004**	0.470
		Control group (*n* = 12)	405.2 ± 62.0	361.8 ± 60.1	0.003**	0.480
	Go Errors	Training group (*n* = 11)	1.2 ± 1.3	0.3 ± 0.6	0.078	0.340
		Control group (*n* = 12)	0.9 ± 1.3	0.8 ± 1.3	0.783	0.060
	No‐Go Errors	Training group (*n* = 11)	3.4 ± 3.1	2.2 ± 2.7	0.384	0.170
		Control group (*n* = 12)	3.3 ± 3.1	2.5 ± 2.5	0.395	0.170

*Note*: Reaction times (in ms) are presented as median ± SD, while numbers of errors are presented as mean ± SD. * : p < 0.05, ** : p < 0.01

### 3.2 TOJ Task

The PSS values obtained before and after the intervention are shown in Table 2. No significant changes were found on day 5 in either the training (*p* = 0.814) or control group (*p* = 0.754).

**TABLE 2:** Point of subjective simultaneity (PSS) values on the temporal order judgment task.

	Day 1	Day 5	*p*	*r*
Training group	−35.9 ± 45.3	−35.0 ± 46.9	0.814	0.040
Control group	−44.4 ± 62.1	−34.7 ± 38.4	0.754	0.060

PSS values (in ms) are presented as mean ± SD.

## 4 Discussion

This study examined the effects of motor training with the visual modality on subsequent task performance using the tactile modality and possible changes in modality dominance with training. Three days of training on a visual Go/No‐go task significantly reduced RTs in both the visual and tactile conditions, but this training did not influence modality dominance as measured by the PSS. These results suggest that visual Go/No‐go training also improves tactile Go/No‐go task performance without influencing modality dominance.

Previous studies using the Go/No‐go task revealed significantly shorter RTs with repeated practice (Benikos, Johnstone, and Roodenrys 2013; Sugawara et al. 2013), especially when the task was of low difficulty (Benikos, Johnstone, and Roodenrys 2013). Additionally, practice on a visual Go/No‐go task significantly reduced the time interval from the first visually evoked field potential peak to peak activity in the posterior parietal cortex (PPC), which is involved in response selection, and the time from PPC activity to EMG activity onset, suggesting improved processing speed in the PPC and transmission speed from the PPC to primary motor cortex (Sugawara et al. 2013). These findings suggest that task practice induces neuroplastic changes in the task‐related cortical regions. We speculate that training for 3 days on the visual Go/No‐go task also induced neuroplastic changes in cortical areas related to visual information processing and motor execution, especially the PPC, which integrates inputs from multiple sensory modalities, including visual, auditory, somatosensory, eye position, eye movement, and vestibular information (Andersen 1997). In addition, the IFG and preSMA are multimodal regions involved in motor control functions (Osada et al. 2019). Based on these studies, the training effect of the visual condition may have modulated the neural activity in IFG, preSMA, and PPC, thereby reducing RT in the tactile condition as well. Furthermore, the performance of the stop signal task, a stimulus‐response task similar to the Go/No‐go task, has been shown to be influenced by the sensory modality (Markiewicz et al. 2023; Friehs et al. 2024). The results of the present study are therefore considered to be valid. On the other hand, the function was improved in the tactile condition of the control group. This may be due to the influence of the 100 Go/No‐go task performed before training. Therefore, in this study, the change rate was calculated and compared between conditions. The results showed significant differences between groups, suggesting that the training was effective.

The PSS was shifted in the visual direction by training in accordance with a previous study (mean ± standard error mean: −10.3 ± 6.7 ms) (Spence et al. 2003), but the difference was greater in the present study (mean ± standard deviation: −35.9 ± 45.3 ms). This may reflect differences in the visual and tactile stimulation parameters such as duration, intensity, and stimulus site. In contrast, the time from each stimulus to PPC activity was reported to be ∼170 ms for visual stimuli (Sugawara et al. 2013) and 25–100 ms for tactile stimuli (Inui et al. 2004; Popescu et al. 2013). Because visual information takes longer to process, the PSS is predicted to shift in the visual direction with training. However, the PSS did not change between days 1 and 5 in either group, possibly because the Go/No‐go task and the TOJ require distinct neural processes (Spence et al. 2003; Spierer et al. 2013). The TOJ examines the detection of temporal asynchrony for different sensory modalities, while the Go/No‐go task (the training task in this study) does not require temporal discrimination as the response is inhibited antecedently (Spierer et al. 2013). The TOJ was used to assess the superiority between the visual and tactile modalities because it was reported to change with sensory exposure in previous studies (Harrar and Harris 2008; Vroomen et al. 2004). However, these previous studies exposed participants to two types of temporally asynchronous sensory stimuli (Harrar and Harris 2008; Vroomen et al. 2004), whereas in the present study, training was conducted using a Go/No‐go task with only one type of sensory stimulus in each session. Additionally, the training effect has two major aspects: the effect on the trained task itself and the transfer effect to a nontrained task (Zhao, Wang, and Maes 2020). Zhao, Wang, and Maes (2020) concluded that the training effect was caused by the trained task itself and transferred only to a similar task. A previous TOJ study using visual and tactile stimuli revealed that PSS was biased toward the visual modality during the preintervention phase, suggesting inherent visual dominance (Harrar and Harris 2008). We also hypothesized that PSS would shift to visual dominance after training. However, this may not occur if visual dominance is inherent (Harrar and Harris 2008), as a further bias would be less likely due to a ceiling effect.

This study has several limitations. First, the cortical activities of the multimodal regions were not measured before and after training. We suggest that neuroplastic changes in multimodal regions, such as IFG, preSMA, and PPC, allow for the observed transfer effect, but this remains to be proven directly using electroencephalography and fMRI. Therefore, it is possible that brain activity measurements would be required in future studies to confirm the actual activity modulation in these regions. Second, it is still unknown whether this effect can occur in the retention. Further investigations need to measure at another time point such as the next day. Third, the number of subjects in this study was small. However, the cross‐modality transfer effects were clearly observed by Go/No‐go training using the visual modality, and the results of this study are valid. To further confirm these results, we would like to increase the number of subjects and conduct more experiments to measure brain activity in the future. Third, this study did not discuss the speed‐accuracy tradeoff. However, in the visual Go/No‐go task, the number of errors decreased in both groups, but the RT decreased only in the training group. On the other hand, no change in the number of errors was observed in the tactile Go/No‐go task. Therefore, the influence of the speed‐accuracy tradeoff is small in this study.

## 5 Conclusion

These results indicate that motor training with visual guidance can improve performance on the same task guided by tactile stimuli, possibly due to neuroplastic changes in the multimodal association cortices.

## Author Contributions

**Maiko Hori**: conceptualization, methodology, investigation, validation, visualization, writing–original draft, formal analysis. **Sho Kojima**: conceptualization, methodology, formal analysis, supervision, project administration, writing–review and editing, writing–original draft, visualization, funding acquisition. **Hideaki Onishi**: conceptualization, methodology, funding acquisition, writing–review and editing, project administration.

## Ethics Statement

This study was conducted in compliance with the Declaration of Helsinki principles, and the study protocol was approved by the Ethics Committee of Niigata University of Health and Welfare (18235).

## Conflicts of Interest

The authors declare no conflicts of interest.

### Peer Review

The peer review history for this article is available at https://publons.com/publon/10.1002/brb3.70309.

## Data Availability

Data will be made available on request.
